# Effectiveness of a group intervention using pain neuroscience education and exercise in women with fibromyalgia: a pragmatic controlled study in primary care

**DOI:** 10.1186/s12891-022-05284-y

**Published:** 2022-04-04

**Authors:** Paula B. Areso-Bóveda, Julia Mambrillas-Varela, Bárbara García-Gómez, José Ignacio Moscosio-Cuevas, Jesús González-Lama, Eva Arnaiz-Rodríguez, María Begoña Arroyo del Barco, Pilar San Teodoro-Blanco

**Affiliations:** 1 Burgos Centre Physiotherapy Unit in Burgos Centre, GAP (Primary Care Management) Burgos (SACYL: Castille and Leon Health Service), C/ José María de la Puente 1, 09006 Burgos, Spain; 2 López Sáiz Health Centre, GAP (Primary Care Management) Burgos (SACYL: Castille and Leon Health Service), C/ José María de la Puente 1, 09006 Burgos, Spain; 3 Fuensanta Health Centre, Córdoba-Guadalquivir Primary Care District (SAS: Andalusian Health Service), C/ Núñez de Balboa 2, 14010 Córdoba, Spain; 4 Group-Program Communication and Health -GPCyS- (semFYC), c/ Diputació, 320 Bajo, 08009 Barcelona, Spain; 5 Clinical Management Unit in Cabra, Matrona Antonia Mesa Fernández Health Centre, South Córdoba Health Management Area (SAS: Andalusian Health Service), Avda. Góngora s/n, 14940-Cabra, Córdoba, Spain; 6 Prevention and Health Promotion Program -PAPPS- (semFYC), c/ Diputació, 320 Bajo, 08009 Barcelona, Spain; 7 Clinical Epidemiology Research Group in Primary Care (GICEAP), Maimónides Biomedical Research Institute of Córdoba (IMIBIC), Reina Sofía University Hospital / University of Córdoba, Avda. Menéndez Pidal s/n, 14004 Córdoba, Spain; 8 San Agustín Physiotherapy Unit, C/ Bonifacio Zamora de Usabel, 09001 Burgos, Spain; 9 José Luis Santamaría Physiotherapy Unit, C/ Lazarillo de Tormes, 09006 Burgos, Spain; 10 Group-Program Communication and Health -GPCyS- (socalemFYC), C/ Veinte de Febrero 6, 47001 Valladolid, Spain; 11 Healthcare Ethics Committee in the Burgos University Hospital, Avda. de las Islas Baleares 3, 09006 Burgos, Spain

**Keywords:** Central nervous system sensitization, Chronic pain, Fibromyalgia, Neurosciences, Pain measurement, Patient education

## Abstract

**Background:**

Very positive effects have been described in the application of pain neuroscience education (PNE) to chronic pain and migraine. However, there are few data on the applicability of this therapeutic approach in actual clinical practice in a primary care (PC) setting. The aim of this study was to explore the efficacy in fibromyalgia (FM) of an intervention based on PNE and exercise compared to treatment as usual (TAU).

**Methods:**

Pragmatic nonrandomised controlled trial set in 5 healthcare centres and one physiotherapy centre in PC. Fifty-three women with FM (2010 American College of Rheumatology Diagnostic Criteria for Fibromyalgia) were studied, 35 in the intervention group (IG) and 18 in the control group (CG). The women in the IG were interviewed individually and then received 6 weekly sessions plus one review session (1 month later): those in the CG received their TAU. The subject assignation to the CG or the IG was determined according to their availability to attend the sessions. They all filled in several questionnaires (prior to and 1 year after the intervention) to evaluate the impact of FM in their daily lives, catastrophism, anxiety and depression, severity and impact of pain in daily personal performance and functional capacity.

**Results:**

The reductions (improvements) in the scores of all tests (baseline-final) were greater in the IG (*p* < 0.05) when adjusted for age and baseline values, with moderate or high effect size. After 1 year, 20% (CI − 1 to 42%) more women in the IG, compared to the CG, had a FIQ score < 39 (mild functional impairment). 17/38 (49%) women in the IG no longer met FM criteria at the end of follow-up.

**Conclusions:**

An intervention based on PNE and exercise in patients with FM is feasible and seems effective in PC.

**Trial registration:**

The study was retrospectively registered at ClinicalTrials.gov (Trial Registration NCT04539171), on 04/09/2020.

## Background

Fibromyalgia (FM) is a complex condition that includes persistent multi-focal pain and hyperalgesia, together with tiredness, sleep and cognitive alterations and other effects [1], with a potentially-serious impact in the quality of life of those who suffer from this condition [2, 3].

Over the last 20 years, the definition of FM has been extended, despite the lack of specific diagnostic tests or pathognomonic findings [4]. This has hampered its treatment which, until now, has consisted in mitigating its symptoms with drugs [5] or even using invasive procedures [6], since no specific treatment exists [4, 7, 8]. The latest recommendations of the EULAR (European League Against Rheumatism) for treating patients with FM indicate that exercise is the only recommended therapeutic option that is strongly supported by evidence. If this fails, other individualised alternatives should be explored such as psychological and pharmacological treatments (for severe pain or sleep alterations) and/or multi-modal rehabilitation programs (in the case of severe disability) [8].

Although they have achieved modest results, several studies have evaluated the efficacy of various non-pharmacological therapies in the treatment of chronic pain syndromes, such as cognitive-behavioural therapy, physical exercise, mindfulness, multidisciplinary approaches, amygdala retraining [9–12] and psychoeducational interventions [13].

In recent years, abundant evidence has been obtained regarding the implication in FM of an abnormal cerebral process primarily mediated by the Central Nervous System (CNS) that forms part of the so-called Central Sensitization Syndromes [4, 14]. Aided by advances in neuro-imaging [15], this concept of pain experience, which differs from nociception, defends the leading role of the brain in the intensity and perception of pain, leaving the relationship with body tissues in second place [16]. In this setting, we find a permanently “hyperalert” nervous system that amplifies sensory stimuli, accompanied by a comprehensive autonomic and/or neuro-endocrine motor cortex. This approach has led to the development of Pain Neuroscience Education (PNE), which proposes that understanding the neurobiological mechanisms involved in the experience of pain reduces the cerebral perception of threat [16]. Achieving a change in the cognition of, and the attitudes related to, the pain process would also moderate the activation of motor, sympathetic and neuro-endocrine protection mechanisms [17].

Very positive effects have been described as regards the application of this therapeutic approach to chronic pain [18–22] and migraine [23, 24], with clinical and functional improvements in patients, including FM sufferers, both as the main therapy [25, 26] and as part of a multidisciplinary approach [27, 28]. Even in the current pandemic due to SARS-CoV-2 infection, some authors such as A. Goicoechea or P. Garner have proposed its use in patients with long COVID-19 due to the similarities of this entity with FM and other central sensitisation syndromes [29, 30]. Nevertheless, there is little data regarding the applicability of this method in real clinical practice in a primary care (PC) setting or of its medium- and long-term effectiveness. For this reason, we undertook a pragmatic study to explore, prospectively, the effectiveness of a double PNE intervention, at group and individual levels, coupled with conscious movement techniques, as compared with treatment as usual (TAU), in PC.

## Methods

### Study design and selection criteria

A pragmatic nonrandomised controlled trial was carried out in 5 urban health centres in Burgos assigned to the Burgos Centro Physiotherapy Unit. The Transparent Reporting of Evaluations with Nonrandomized Designs (TREND) guidelines have been followed in presenting the results [31].

Each PC team from the 5 participating Health Centres received a clinical session in which the PNE model was explained, and the referral to the PC Physiotherapy Units of patients who met the ACR 2010 diagnostic criteria for FM was requested. Primary care physiotherapists are integrated in the Spanish PC system and provide individual and group care to the population.

Patients of 18 years of age or older who met the ACR 2010 diagnostic criteria for FM (American College of Rheumatology) [1] were recruited using opportunistic sampling (a non-probability sampling that offered voluntary participation to an accessible sample of the study target population). Patients with incapacitating mental diseases or intellectual deficits that could hinder follow-up of the intervention were excluded. The candidates were included in a waiting list and were then incorporated into the intervention and control groups according to the patients’ possibilities of participating in the sessions. Therefore, the control group (CG) was composed of those women who met the FM criteria but who, for different reasons, postponed their participation in the sessions. The patients in the CG were offered the possibility of subsequently participating in the intervention after the end of the study if they so wished. No limitation was applied to any treatment during the study.

### Initial session for patients in the control and intervention groups

All patients included in the study attended an initial session in which the degree of fulfilment with the FM criteria, including the number of pain areas (WPI) and the symptom severity (SS), was assessed. Patients were provided with various questionnaires to fill in themselves: impact of FM in daily life (S-FIQ), catastrophizing (PCS), anxiety and depression (HAD), severity and impact of pain in daily performance (BPI), and functional capacity (HAQ). These questionnaires were repeated 1 year later, both in the CG and in the intervention group (IG), to evaluate the changes in these factors over this time period. In addition, social-economic variables and personal and family histories potentially related to the condition were collected.

### Treatment as usual (control group)

The patients in the CG received no additional therapy beyond the treatment they were undergoing, although they were informed that in the future, they could receive the group intervention if they wished to. In Spain, the usual treatment for patients with FM is mainly pharmacological and adjusted to the symptomatic profile of each individual subject, mostly including antidepressants, antiepileptics and opioid and non-opioid analgesics. Exercise tailored to the patients’ physical limitations are usually recommended based on recommendations of scientific societies summarised in a document issued by the Spanish Ministry of Health [32].

### Study intervention

The patients in the IG were divided into 4 groups of 9, 11, 12 and 13 women. Two of these groups were attended by a Family doctor and a physiotherapist, and the other two by a nurse and a physiotherapist, who were responsible for them during the intervention. All workers carried out the interventions during their working day, together with their normal activities: no additional personnel were recruited for this study.

A first individual interview was conducted, lasting between 60 and 90 min, in which through active listening the sociodemographic and health data, history of personal and family pain, and their opinions and beliefs about FM and its impact were collected.

Subsequently, a group intervention was carried out consisting of 6 consecutive weekly sessions each lasting 2 h, as described by Barrenengoa-Cuadra et al. [25]. In these, the latest knowledge regarding the neurophysiology of pain was explained, following pre-established guidelines and supported with a PowerPoint presentation. One month after the sixth session, a review session was carried out.

During these sessions, concepts such as pain-injury, necrosis-apoptosis, sensation-perception, nociception-pain, proprioception, brain mapping, neuromatrix, efference copy, pain memory, placebo-nocebo, hypervigilance, central sensitization, neuroplasticity, compensation systems, beliefs and memory learning, based on PNE, and specifically on the work of Arturo Goicoechea, David Butler and Lorimer Moseley, were addressed [16, 24].

At the same time, in all of these sessions, the physiotherapists explained how to perform exercises aimed at body awareness and attention via gradual exposure to movement. In particular, the fifth session was dedicated exclusively to exercises. The aim was to promote body movements via conscious breathing, with different exercises involving tactile sensations (proprioception), flexibility and strength. Games were used to generate spontaneous movements, favouring coordination and balance, to reinforce the theoretical concepts. Each exercise session was divided into three parts:

- Warm-up exercises to promote joint mobility, conscious breathing and self-massage to improve proprioception and body awareness.
- Mobility, coordination, strength and balance exercises, accompanied by games or music. Children’s games or ball games encourage spontaneous movement and contact between patients.
- Return to calm or relaxation techniques.

In each session, patients received a summary of the session and reading matter supporting what they had learned. Great care was taken over the language used in the explanations, to prevent it acting as a nocebo and to avoid catastrophizing [27, 28, 33].

### Data collected during the study

- ACR 2010 diagnostic criteria for FM: we used the Widespread Pain Index (WPI), to evaluate the number of areas in which the patient had experienced pain during the previous week (the score that varies between 0 and 19), and the Symptom Severity (SS), to quantify the severity of the symptoms (the score, that varies from 0 to 12, is the sum of fatigue, unrefreshed sleep and cognitive alteration plus the symptoms habitually suffered by the patient). As in the other scales described below, higher scores indicate worse results for the patients.
- Impact of FM in daily life: physical functions, global impact and symptom severity were evaluated using the Spanish version of the Fibromyalgia Impact Questionnaire (S-FIQ: score between 0 and 100). As described in other studies [34], we used 3 cut-off points to compare the two study groups: ≥20% reduction on the FIQ total score from baseline to 1 year later; ≥50% reduction on the FIQ total score from baseline to 1 year later; and number of patients crossing a cut-off point (reaching no worse than mild functional impairment; FIQ total score < 39).
- Catastrophizing: this was evaluated with the Spanish version of the Pain Catastrophizing Scale (PCS: score between 0 and 52) [35].
- Anxiety and depression: we used the Hospital Anxiety and Depression Scale (HAD), which consists of two sub-scales, one to evaluate anxiety and the other for depression (in both, the score runs from 0 to 21) [36].
- Severity and impact of pain in the patient’s daily life: we used the Brief Pain Questionnaire (BPI-sf), which consists of two parts, one to evaluate pain intensity, and the other to assess its influence in different aspects of life during the previous week (the total score runs from 0 to 70).
- Functional capacity: we used the Spanish version of the Health Assessment Questionnaire (HAQ), which evaluates the difficulty in performing different activities of daily life (score from 0 to 3).
- Other metrics: age, marital status, educational level, employment situation, history of mistreatment, abuse, traumatic accidents, abortions, chronic dysmenorrhea, chronic headaches and pain during infancy/adolescence, years since onset of polymyalgia, years since FM diagnosis and age at the onset of symptoms, primary home caregiver, and history of chronic pain in household/family members, broken family and unexpected deaths in household/family members.

### Statistical analysis

The decrease in FIQ scores (using the 3 cut-off points described above) at the end of the study as compared with baseline was chosen as the primary outcome measure. As this was a pragmatic and feasibility study a formal sample size calculation was not determined [37]: all patients referred to our Physiotherapy Unit who met the study selection criteria were invited to participate.

Numerical variables were summarized with means ± standard deviations and 95% confidence intervals (95% CI). For summarized categorical variables, percentages were calculated. To compare changes in means inside groups and between groups, we applied the paired and unpaired Student’s t-test, respectively; for proportions chi-square tests were used. The intergroup comparison of the changes in FIQ and other questionnaire scores were assessed by analysis of covariance with adjustments for baseline values and age. The effect size was evaluated using Cohen’s d (d = 0.20 small, d = 0.50 medium, and d = 0.80 large effect sizes, respectively) [38]. Analyses were performed on a per protocol basis. The results are presented with corresponding 95% confidence intervals (CIs). The significance level was set at *P* < 0.05. Data analysis was performed by using the SPSS v24 statistical package.

## Results

**Fig. 1:**
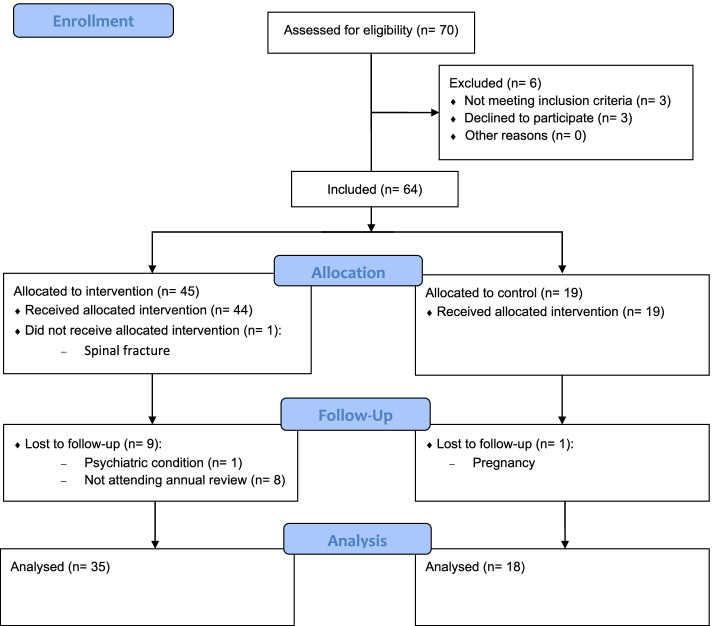
Flow diagram

Between January 2018 and October 2019, 4 group courses were organized for a total of 45 patients who met FM criteria (IG). In parallel with this, data were collected from 19 women who did not receive the intervention (CG). Of these 64 women, 53 answered the initial and final questionnaires (the latter 1 year after the initial interview), 35 from the IG and 18 from the CG (Fig. 1).

The baseline characteristics of the 53 women included in the analysis are shown in Table 1. The average age of the women in the CG was greater than that in the IG (64 vs 57 years). In general, the IG included a greater proportion of single women, women with secondary/higher education, with paid work, with a history of significant pain during infancy and with a higher score for severity of symptoms than did the CG.

**Table 1:** Baseline characteristics (*n* = 53)

Variable	Control (*n* = 18)	Intervention (*n* = 35)	*p*
Age, years	64.1^a^ ± 9.0^b^	57.4 ± 9.1	*0.014*
Marital status			*0.021*
Married/with partner	11 (61%)	24 (69%)	
Divorced/separated	3 (17%)	4 (11%)	
Widow	3 (17%)	0	
Single	0	7 (20%)	
No Answer	1 (6%)	0	
Level of education			*0.06*
No education	1 (6%)	0	
Primary education	12 (66%)	15 (43%)	
Secondary education	4 (22%)	14 (40%)	
Higher education	0	6 (17%)	
No Answer	1 (6%)	0	
Work situation			*0.022*
Retired	9 (50%)	5 (14%)	
Permanent disability	0	1 (3%)	
Home carer (unpaid)	2 (11%)	10 (28%)	
Unemployed	3 (17%)	2 (6%)	
Working	4 (22%)	17 (49%)	
History of mistreatment	5 (28%)	4 (11%)	*0.13*
History of abuse	0	1 (3%)	*1.0*
History of traumatic accidents	2 (11%)	8 (23%)	*0.47*
History of abortions	0	4 (11%)	*0.29*
History of chronic dysmenorrhea	4 (22%)	12 (34%)	*0.53*
History of chronic headaches in infancy/adolescence	4 (22%)	8 (23%)	*1.0*
History of significant pain during infancy	3 (17%)	22 (63%)	*0.003*
Polymyalgia: years since onset of symptoms	25.5 ± 19.0	30.4 ± 17.1	*0.36*
FM: years since diagnosis	10.13 ± 8.7	8.4 ± 8.5	0.51
Age at onset of symptoms, years	25.5 ± 19.0	30.4 ± 17.1	0.36
Primary carer	15 (83%)	27 (77%)	0.24
History of chronic pain in household/ family members	18 (100%)	32 (91%)	0.54
History of broken family	0	2 (6%)	1.0
History of unexpected death in household/ family members	5 (28%)	14 (40%)	0.76
WPI	11.76 ± 3.0	11.74 ± 3.7	0.98
SS	8.0 ± 1.8	9.1 ± 1.5	0.028
i-BPI-sf	25.17 ± 6.9	24.5 ± 7.1	0.76
a-BPI-sf	47.4 ± 10.9	44.9 ± 16.0	0.49
FIQ	59.5 ± 19.1	67.6 ± 14.4	0.09
A-HAD	12.6 ± 3.7	11.9 ± 3.3	0.53
D-HAD	10.4 ± 4.4	9.6 ± 4.0	0.47
T-HAD	23.1 ± 6.7	21.5 ± 6.5	0.43
HAQ	1.2 ± 0.8	1.3 ± 0.6	0.50
PCS-SP	28.4 ± 9.9	26.4 ± 11.6	0.52

*FM* fibromyalgia, *WPI* Widespread Pain Index, *SS* symptom severity prior to the intervention, *i-BPI-sf* pain intensity sub-scale of the Brief Pain Inventory, *a-BPI-sf* impact on life sub-scale of the Brief Pain Inventory, *FIQ* Fibromyalgia Impact Questionnaire, *A-HAD* anxiety sub-scale of the Hospital Anxiety and Depression (*HAD*) Questionnaire, *D-HAD* depression sub-scale of the Hospital Anxiety and Depression (*HAD*) Questionnaire, *T-HAD* total score (anxiety + depression) of the Hospital Anxiety and Depression (*HAD*) Questionnaire, *HAQ* functional capacity, *PCS* Pain Catastrophizing Scale, Spanish version (*PCS-SP*)

^a^Average

^b^Standard deviation

Table 2 shows the variations observed in the different variables between the baseline scores and the scores after 1 year, both within each group and between the IG and the CG. In the intervention group, a reduction (improvement) was observed in the scores of all variables at the one-year follow-up. In the control group, a worsening (increase in score) in symptom seriousness (SS), impact of FM on daily life (FIQ) and functional capacity (HAQ) was noted. Moreover, the improvement observed in the scores of all variables (comparing the differences between the scores obtained for each variable in IG and CG) was greater in the IG than in the CG, with all differences reaching statistical significance (*p* < 0.05), after adjusting for age and baseline scores. In addition, the effect sizes of the differences between the intervention and the control groups were large for pain and FIQ total score, and medium for the remaining dimensions (Table 2).

**Table 2:** Variation of the values in the two study groups

	Control (*n* = 18)			Intervention (*n* = 35)			Difference, intervention-control (95% CI)^a^	Cohen’s d effect size (95% CI)
	Pre	Post	Diff. Post-Pre	Pre	Post	Diff. Post-Pre		
WPI	11.5^b^ ± 3.1^c^	10.6 ± 3.8	−0.9 ± 3.9	11.9 ± 3.7	7.1 ± 4.2	−4.6 ± 4.0	−3.7 (from − 6.6 to −2.0)	0.9 (0.3 to 1.53)
SS	7.9 ± 1.8	8.3 ± 1.9	0.3 ± 2.4	8.9 ± 1.6	6.0 ± 2.4	− 3.1 ± 2.5	−3.3 (from − 4.6 to − 2.0)	1.4 (0.7 to 2,0)
i-BPI-sf	25.21 ± 6.7	22.7 ± 6.8	− 2.5 ± 6.9	25.2 ± 6.7	17.1 ± 9.4	−7.5 ± 8.0	−5.0 (from −9.4 to − 0.5)	0.7 (0.1 to 1.2)
a-BPI-sf	47.1 ± 10.7	43 ± 14.3	− 4.4 ± 12.8	45.5 ± 14.8	26.9 ± 16.9	− 18 ± 17.4	−13.6 (from − 22.9 to − 4.2)	0.9 (0.3 to 1.4)
FIQ	59.6 ± 18.6	62.0 ± 22.7	2.5 ± 20.8	68.3 ± 14.0	47.6 ± 25.1	−20.0 ± 24.9	−22.5 (from − 36.2 to − 8.8)	1.0 (0.4 to 1.6)
A-HAD	12.3 ± 3.9	11.1 ± 3.3	−1.6 ± 2.9	12.0 ± 3.6	8.9 ± 4.2	−3.1 ± 4.6	− 1.6 (from − 4.0 a − 0.9)	0.4 (− 0.2 to 1.0)
D-HAD	10.2 ± 4.4	9.3 ± 4.2	−1.2 ± 3.1	9.8 ± 4.0	6.5 ± 5.1	3.1 ± 5.1	−1.9 (from − 4.6 to − 0.7)	0.4 (− 0.2 to 1.0)
T-HAD	22.5 ± 6.9	20.3 ± 6.3	− 2.7 ± 5.4	21.6 ± 6.8	15.3 ± 8. 3	−6.2 ± 9.2	−3.5 (from − 8.2 to − 1.3)	0.4 (−0.2 to 1.0)
HAQ	1.2 ± 0.8	1.3 ± 0.8	0.1 ± 0.6	1.4 ± 0.7	0.9 ± 0.7	−0.4 ± 0.7	−0.5 (from − 0.9 to − 0.1)	0.7 (0.1 to 1.3)
PCS-SP	28.8 ± 9.8	23.8 ± 9.4	− 4.7 ± 6.7	28.3 ± 12.2	15.9 ± 12.4	− 11.3 ± 11.6	−6.6 (from − 11.7 to − 1.6)	0.7 (0.1 to 1.2)

*Pre* prior to starting the intervention (initial visit), *Post* 1 year after finishing the intervention (final visit), *WPI* Widespread Pain Index, *SS* Symptom severity prior to the intervention, *i-BPI-sf* pain intensity sub-scale of the Brief Pain Inventory, *a-BPI-sf* impact on life sub-scale of the Brief Pain Inventory, *FIQ* Fibromyalgia Impact Questionnaire, *A-HAD* anxiety sub-scale of the Hospital Anxiety and Depression (*HAD*) Questionnaire, *D-HAD* depression sub-scale of the Hospital Anxiety and Depression (*HAD*) Questionnaire, *T-HAD* total score (anxiety + depression) of the Hospital Anxiety and Depression (*HAD*) Questionnaire, *HAQ* functional capacity, *PCS* Pain Catastrophizing Scale, Spanish version (*PCS-SP*)

^a^After adjusting for age and baseline values

^b^Average

^c^Standard deviation

As shown in Table 3, 17 (49%, CI: 33–64%) of the women in the IG no longer fulfilled the FM criteria 1 year later, according to the ACR 2010 classification.

**Table 3:** Respondents according to cut-off points (FIQ and ACR 2010) at 12 months

Cut-off points	12 months after starting the intervention		
	Control (*n* = 18) *n* (%)	Intervention (*n* = 35) *n* (%)	Difference, intervention-control (%) (95% CI)
Negativisation of ACR 2010 diagnostic criteria	0 (0)	17 (48.6)	*48.6 (from 32.0 to 65.1)*
Reduction of FIQ ≥ 20%	4 (22.2)	19 (54.3)	*32.1 (from 6.7 to 57.4)*
Reduction of FIQ ≥ 50%	1 (05.6)	9 (25.7)	*20.1 (from 2.2 to 38.1)*
Score < 39	2 (11.1)	11 (31.4)	20.3 (from − 0.8 to 41.5)

*ACR* American College of Rheumatology, *FIQ* Fibromyalgia Impact Questionnaire

Regarding the evaluation of the impact of fibromyalgia, more women in the IG showed a reduction ≥20% and ≥ 50% between the baseline FIQ score and the final score. Also, at the end of the study, 2 (11%, CI: 3–33%) and 11 (31%, CI 19.48%) of patients achieved a FIQ score < 39 (mild functional impairment) in the CG and the IG, respectively, although this difference was not statistically significant (Table 3).

## Discussion

Our study shows that a group intervention based on PNE, preceded by an active-listening interview and accompanied by exercises of increasing intensity, can improve, among other factors, the severity of symptoms, the functional capacity and the impact of pain in the daily life of women with FM. This improvement was sustained for 1 year.

Up to now, few studies have been published on patients with FM carried out in PC to evaluate PNE-based interventions. Our results corroborate those obtained in an uncontrolled before-after study where a similar PNE methodology was used, but without the addition of exercise [25]. As in that study, we used a modified form of classical PNE, incorporating the hypothesis of learned cerebral evaluation error. This hypothesis implies an original approach since it implicates an unconscious learning process in the aetiopathogenesis of this disease, removing blame from the subject. It focusses on the unconscious transmission of knowledge in order to unlearn the painful experience associated with the learned cerebral evaluation error, which is reinforced by the current alarmist culture, model copying and instructions from experts [24].

The results obtained in our study are comparable to the best results reported in published controlled studies that used various interventions based on PNE [26, 34, 39]. Van Ittersum et al. [26] concluded that supplying written information on the physiology of pain, followed by a motivational telephone call to resolve doubts, was not effective in patients with FM in terms of modifying the impact of pain in daily life, of improving feelings related to pain (catastrophizing) nor in perceptions regarding the condition. In the study by van Oosterwijck et al. [39], an intervention consisting of 2 individual PNE sessions, each of 30 min, achieved a reduced degree of anxiety in the short term and longer-term improvements in vitality, functionality, mental health and in the general perception of health; however, the pressure pain thresholds remained unchanged. The EFFIGACT study, the only one carried out in PC, showed that the group that received the PNE intervention (8 sessions of 2.5 h each with groups of 10 to 15 patients) experienced a greater increase in their overall functional state than the group treated with drugs or the CG; they also reported improvements in the pain catastrophizing scale, acceptance of pain, subjective pain, quality of life, and anxiety and depression, with medium-sized effects in a majority of cases.

These differences may be due not only to the particular design of each study, but also to the type and duration of the PNE sessions. In a recently-published study that evaluated the effects of different dosages of PNE in 3 centres specializing in FM in Spain [40], it was found that higher dosages (6 sessions, each of 45 min) produced a larger improvement in pain severity at three-month follow-up than other dosages of PNE (2 sessions of 45 min or 6 sessions of 15 min) and biomedical education (2 sessions of 45 min); however, PNE (regardless of its duration) was not superior to biomedical education in the central nociceptive processing, disability, or psychological variables in patients with FM.

In addition, in all our sessions, the physiotherapists explained how to carry out different exercises, and the fifth session was dedicated exclusively to exercises. It should be emphasised that the physiotherapist is the most qualified professional to carry out the exercise programme of the intervention, so it is strongly recommended that these professionals are the ones who teach and direct the exercises. In this sense, the results of a study carried out in a hospital environment have just been published, following the recommendations of the EULAR [8], which establishes education and physical exercise (carried out by physiotherapists) as the initial management for FM. This study concluded that a multidisciplinary intervention (including PNE and exercise) achieved moderate to large effect at six-weeks, when compared with the control group (TAU), for functional impairment, anxiety, kinesiophobia, perceived competence, and positive reappraisal in patients with FM [41].

Another difference in our intervention, compared to that used in the aforementioned studies, is that before starting the group sessions, each patient was interviewed individually, using active listening to aid in the verbalization of their experience of the disease. During these interviews, we could perceive certain characteristics that could be associated a priori with poorer results in the variables analyzed, such as the expectation of receiving an indemnity (for inability to work or some degree of disability), social/family or labour-related advantages derived from suffering this disease, or a feeling of dissatisfaction with the way in which the health system (including some professionals) had treated them. These aspects deserve to be investigated in ad hoc studies using qualitative research techniques such as focal groups.

As described above, the sessions were delivered in groups of 9–13 subjects. Although theoretically this intervention could be applied to a single subject, it is much more cost-effective to use it in a group of subjects, as proposed in most studies, in the same way that we offer other services such as maternal education, pelvic exercises to improve urinary health, etc. in PC. It is known that there are some limitations in some PC sites to implement group interventions, so it is necessary to make an effort to ask health authorities and PC managers to promote and allocate the necessary resources where they do not exist to offer these activities to the population.

In order to be recruited for our study, patients had to meet the ACR 2010 diagnostic criteria for FM [1]. As in the case reported by Barrenengoa-Cuadra et al. [25], it is noteworthy that in our study almost half of the patients in the intervention group no longer met the criteria for FM 1 year later. As the authors of that study also remarked, we have not found this evaluation criterion in the literature that we have reviewed, so it would be interesting to evaluate its usefulness in following up these patients.

Among the strengths of this study are that it is a pragmatic, controlled trial that evaluates an intervention carried out by a professional multidisciplinary team (physiotherapists, nurses and family doctors) in PC, integrated in the normal activities of this level of care, without requiring additional resources. Although the ideal design for demonstrating the benefits of an intervention is the classical randomised clinical trial, it has the limitations of using strict selection criteria with very clear and complex rules for implementing the intervention, which makes it difficult to replicate in clinical practice. As commented above, some randomised clinical trials have been published exploring the efficacy of the PNE approach. Therefore, we decided to apply this method in a standard clinical setting to demonstrate that it is feasible and compatible with the routine care of other patients. In this sense, we decided to offer the intervention to all potential patients, as we do with other interventions/treatments, and let them decide whether they wanted to be included in the intervention or control group, depending on their current availability to attend the sessions. The selection criteria used in our study were not excessively strict, so the results obtained could be extrapolated to most of patients with FM in PC (high external validity).

Although male patients were not excluded, none were included in the study because no males with FM visited the Physiotherapy Unit during the recruitment period: however, in the studies of patients with FM that we have consulted, it is unusual to find a proportion of male patients greater than 5–10%, which is in line with the known prevalence of this condition. The low drop-out is also noteworthy, despite the sessions being carried out every week during almost 2 months, and with a one-year follow-up period (in most of the clinical trials published, the follow-up was 6 months at most).

All data on the variables studied were self-reported, so, although this was not a blinded trial, it would be difficult for the researchers to have influenced the results. Given the nature of the intervention, it would not be possible for the educators to be unaware of the patients’ groups.

The relatively small size of the sample (especially in the case of the control group) did not allow for subgroup analysis to determine which subgroups of patients could benefit most from this intervention, nor to detect small differences between the groups. Nevertheless, given the magnitude of the differences observed between the two populations in most of the variables studied, it is not likely that the true differences differ greatly from those observed. In addition, the 95% CI is given for all results, so that readers can draw their own conclusions in each case. On the other hand, the distribution of the patients between the intervention and the control groups was determined by their own preferences, which allows us to suppose that there was greater motivation in the intervention group, and this may have influenced the magnitude of the results. However, this is a characteristic of most studies that investigate cognitive-behavioural therapies and is difficult to control for.

As for the non-existence of a control group that received no treatment, which would have made it possible to compare the effect of the intervention with the natural history of FM, it did not seem ethical to us, in a pathology associated with a high degree of physical and psychological suffering, asking patients to abandon any therapy they were receiving. However, given that all the patients continued their usual follow-up by professionals from the same Health Area, it is not expected that there would be relevant differences in the basic treatment received by the patients in both groups: therefore, the differences observed can reasonably be attributed to the intervention studied, especially bearing in mind that they are in line with those described in other studies.

## Conclusions

The results obtained show that this intervention based on PNE and exercise is feasible and effective in patients with fibromyalgia in real practice in PC, possibly the ideal level of care for the management of this type of patients. Group therapy can increase the degree of adherence and the positive responses of peers and can promote and reinforce the assimilation of the changes required in order to unlearn the painful experience associated with the learned cerebral evaluation error. This hypothesis deserves to be tested in an ad hoc study that could also study the efficiency of this group intervention compared with TAU in terms of direct costs (number of consultations in PC, hospitals and emergency departments, medical drugs consumption, etc.) and of indirect costs (days off work, disabilities, etc.).

## Data Availability

The datasets used and analysed during the current study are available from the corresponding author on reasonable request.

## Abbreviations

BPI-sf: Brief Pain Questionnaire
CG: Control Group
CI: Confidence Interval
CNS: Central Nervous System
FIQ: Fibromyalgia Impact Questionnaire
FM: Fibromyalgia
GAP: Primary Care Management
HAD: Hospital Anxiety and Depression
HAQ: Health Assessment Questionnaire
IG: Intervention Group
PC: Primary Care
PCS: Pain Catastrophizing Scale
PNE: Pain Neuroscience Education
S-FIQ: Spanish version of the Fibromyalgia Impact Questionnaire
SS: Symptom Severity
TAU: Treatment As Usual
TREND: Transparent Reporting of Evaluations with Nonrandomized Designs
WPI: Widespread Pain Index
